# *FOXK2* gene expression in cancer: Potential regulatory mechanisms and clinical implications

**DOI:** 10.1016/j.gendis.2025.101951

**Published:** 2025-11-24

**Authors:** Renata Ivo Vasconcelos, Luciana da Torre Carneiro, Raquel Ciuvalschi Maia, Thaís Hancio, Gabriela Nestal de Moraes

**Affiliations:** a Instituto de Bioquímica Médica Leopoldo de Meis, Universidade Federal do Rio de Janeiro (UFRJ), Avenida Carlos Chagas Filho, 373, 2° andar, H2-003, Cidade Universitária, Rio de Janeiro 21 941 599, Brazil; b Programa de Hemato-Oncologia Molecular, Instituto Nacional do Câncer (INCA), Praça da Cruz Vermelha, 23, 6° andar, Centro, Rio de Janeiro 20 230 130, Brazil; c Laboratório Nacional de Biociências (LNBio), Centro Nacional de Pesquisa em Energia e Materiais (CNPEM), Polo II de Alta Tecnologia, R. Giuseppe Máximo Scolfaro, 10000, Bosque das Palmeiras, Campinas, São Paulo 13 083 100, Brazil

**Keywords:** Cancer, Clinical outcomes, FOXK2 transcription factor, Gene alterations, Transcript expression

## Abstract

FOXK2 is a transcription factor known to regulate a wide range of biological processes that are critically involved in determining cell fate. Increasing evidence shows aberrant FOXK2 expression in some tumors, with crucial biological and clinical impacts. It is important to note that the molecular mechanisms contributing to *FOXK2* gene deregulation are poorly understood for most cancers. In this review, we systematically describe the *FOXK2* gene expression profile across distinct tumor types and discuss its potential utility as a prognostic and diagnostic molecular marker. Notably, we explore emerging mechanisms accounting for *FOXK2* deregulation, focusing on genetic and transcriptional modifications, such as gene methylation, mutation and copy number variations.

## Introduction

The *Forkhead box K2* (*FOXK2*) gene is located on chromosome 17q25.3 and consists of 10 exons. *FOXK2* mRNA is widely expressed and undergoes alternative splicing, generating three still unexplored isoforms.^1^ This gene encodes a 660 amino acid protein that belongs to the forkhead superfamily of transcription factors. As such, the FOXK2 structure includes FOX DNA binding, as well as the forkhead-associated (FHA) domain. Seminal studies have demonstrated that FOXK2 plays a crucial role in metabolism, with functions in autophagy,^2^^,^^3^ glucose metabolism,^4^, ^5^, ^6^ nucleotide *de novo* synthesis,^7^ fatty acid oxidation and mitochondrial biogenesis.^8^

Beyond normal physiology, a role in suppressing adenoviral-mediated tumor formation was attributed to FOXK2 in 2010.^9^ Since then, the implications of FOXK2 for cancer have been increasingly explored, with reports pointing to contradictory roles as a tumor suppressor or an oncogene, depending on the tissue of origin (please see^10^, ^11^, ^12^, ^13^, for review articles). Despite these inconsistencies, *FOXK2* gene expression and clinical relevance have not been systematically investigated in human cancer, which hinders direct comparisons across distinct tumor types.

In this review, we address the *FOXK2* gene expression pattern in a wide range of cancer types, highlighting novel information on how it provides prognostic and diagnostic information of clinical utility. Furthermore, we discuss genetic alterations and potential regulation mechanisms of *FOXK2* gene transcription in the context of the emerging functions of FOXK2 in cancer research.

## *FOXK2* gene expression in human cancer

**Figure 1:**
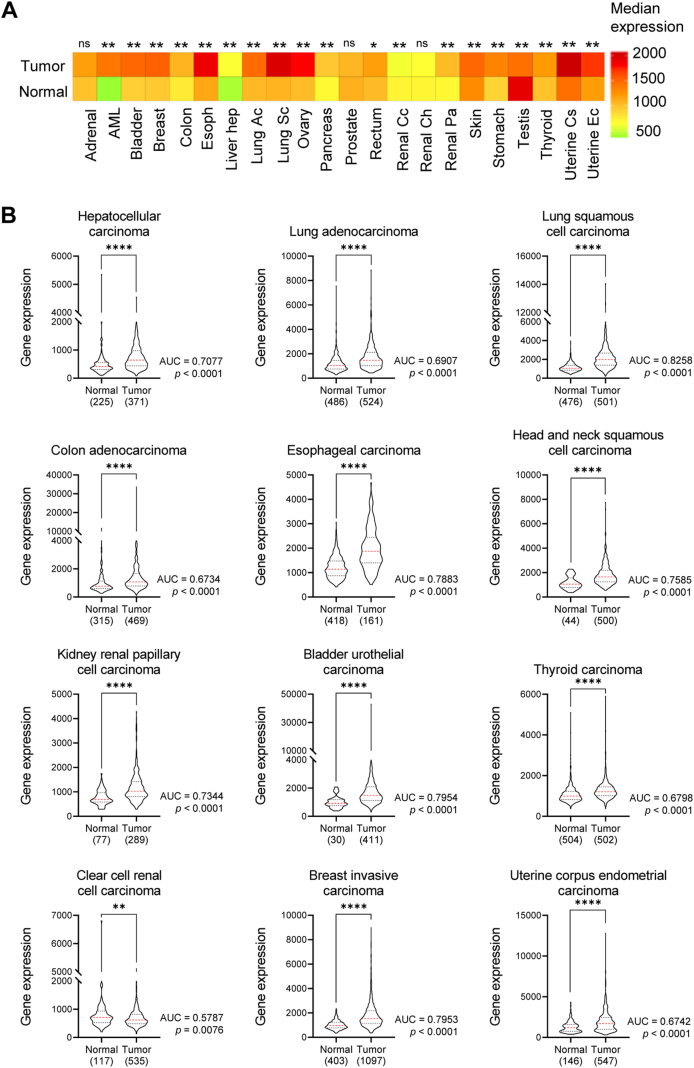
*FOXK2* gene is differentially expressed in tumor versus normal types. **(A)** Heatmap of *FOXK2* gene expression in normal and tumor tissue samples from 22 different tumor types. AML = Acute Myeloid Leukemia; Esoph = Esophageal Carcinoma; Liver hep = Liver Hepatocellular Carcinoma; Lung Ac = Lung Adenocarcinoma; Lung Sc = Lung Squamous Cell Carcinoma; Renal Cc = Renal Clear Cell Carcinoma; Renal Ch = Renal Chromophobe Cell Carcinoma; Renal Pa = Renal Papillary Cell Carcinoma; Uterine Cs = Uterine Carcinosarcoma; Uterine Ec = Uterine Corpus Endometrial Carcinoma. A Mann–Whitney *P* < 0.05 value was used to identify the *FOXK2* gene, which was differentially expressed at least ten times between normal and tumor tissues. **(B)** Violin plots of the *FOXK2* gene differentially expressed between normal and tumor tissue samples from 12 different tumor types. The normal distribution of the samples was evaluated via the D’Agostino & Pearson test, and the cohorts were compared via the Mann–Whitney test. The transcriptome profiling of the samples was processed via the RNAseq technique. Databases: TCGA (The Cancer Genome Atlas) and GTEx (Genotype-Tissue Expression). The receiver operating characteristic (ROC) curves were analyzed and the area under curve (AUC) values with respective *P* values are shown. ns, non-significant; ∗*P* < 0.05; ∗∗*P* < 0.01; ∗∗∗*P* < 0.001; ∗∗∗∗*P* < 0.0001. Data were extracted from the TNM plot platform, and graphs were constructed with GraphPad Prism 8.0.1.

FOXK2 plays a dual role in tumor biology, acting either as an oncogene or a tumor suppressor in different tumor types.^10^ Supporting this, we compared *FOXK2* transcript expression between normal and tumor samples from RNA-seq transcriptome level databases for each tumor type available on the TNMplot.com platform.^14^ From the heatmaps of 22 different tumor types (Fig. 1A) and representative graphs (Fig. 1B; Fig. S1), we found that *FOXK2* transcript levels were significantly higher in most tumors than in non-neoplastic tissues (data available at tnmplot.com). This included hepatocellular (HCC), lung squamous cell (LUSC), esophageal squamous cell (ESCA), head and neck squamous cell (HNSC), kidney renal papillary (KIRP), bladder urothelial (BLCA), thyroid (THCA), uterine corpus endometrial (UCEC), and breast invasive carcinoma (BRCA) as well as lung (LUAD), colon adenocarcinoma (COAD) (Fig. 1B) and other cancer types, including pediatric tumors (Fig. S1). In contrast, clear cell renal cell carcinoma (ccRCC) (Fig. 1B) and testicular germ cell tumor (TGCT) (Fig. S1) exhibited lower *FOXK2* expression levels than normal tissues.

Our finding of *FOXK2* overexpression in a wide range of human cancers seems unexpected at first and could point to an oncogenic function, which contrasts with reports of FOXK2 as a tumor suppressor in most cancer tissues. However, a common feature of cancer is genomic instability, which resulted from defective DNA repair mechanisms and thus accumulated DNA damage.^15^ Notably, FOXK2 has been shown to bind to G/T mismatch regions^16^ and cytosine modifications in DNA,^17^ further implicating its participation in processes involved in DNA modifications and repair. As such, we could argue that the FOXK2 transcription factor might act as a sensor of DNA damage in cancer cells, further activating the DNA repair machinery. Interestingly, we previously demonstrated that the FOXK2 expression is induced at both the protein and mRNA levels in response to chemotherapy-mediated genotoxic stress in breast cancer cellular models.^18^ More recently, this issue was addressed by Chen et al (2020),^3^ who reported that FOXK2 is post-translationally modified by the CHK2 kinase upon cisplatin-induced DNA damage and autophagy activation. Altogether, these findings suggest that *FOXK2* up-regulation in tumors might result from adaptation to abnormal DNA damage originating from replicative stress and genome instability typical of malignant transformation.

## Clinical and biological implications of *FOXK2* gene expression

**Figure 2:**
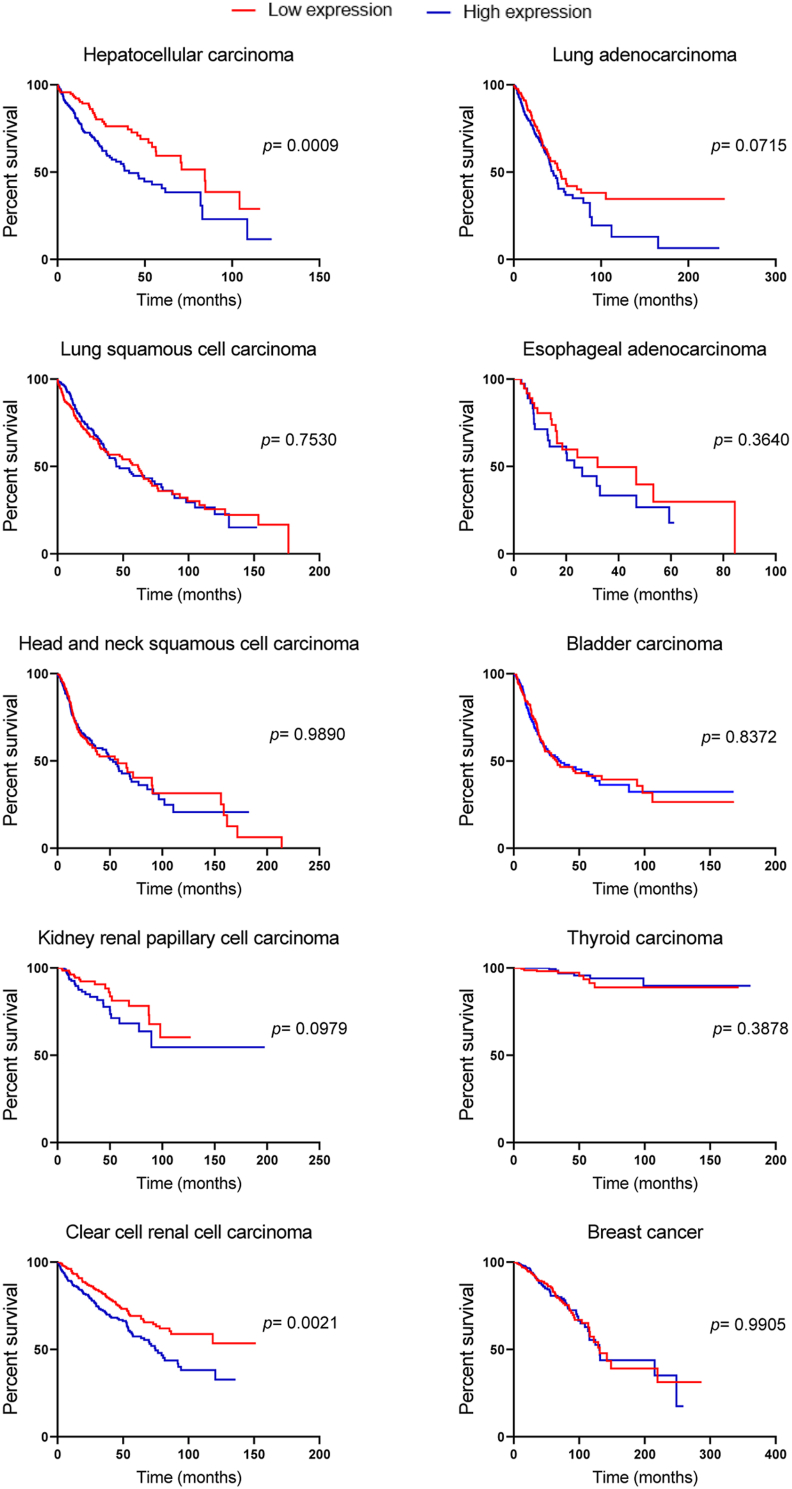
Impact of *FOXK2* gene expression on patient overall survival. Correlation between Kaplan–Meier overall survival curves and low (red line) or high (blue line) FOXK2 gene expression groups in different tumor types are plotted with respective log rank *P* values. *FOXK2* gene expression levels were evaluated using RNAseq data, and the patients were split based on the median values. Gene expression and overall survival data were derived from the GEO (Gene Expression Omnibus), EGA (European Genome-phenome Archive) and TCGA databases. The datasets were extracted from the Kaplan–Meier plotter platform. The graphs and statistical tests were performed with GraphPad Prism 8.0.1.

Based on these findings, we questioned whether high levels of *FOXK2* would have biological and prognostic implications for cancer patients. For this purpose, we extracted data from the Kaplan–Meier plotter platform (data available at kmplot.com)^19^ and correlated *FOXK2* transcript expression with overall survival in some tumor types (Fig. 2). Consistent with elevated *FOXK2* gene expression in HCC tissues, when compared to normal samples (AUC = 0.7077; *P* < 0.0001) (Fig. 1B), lower *FOXK2* expression predicts better overall survival rates for HCC patients (log-rank test *P* = 0.0009) (Fig. 2). These findings corroborate data obtained by other groups regarding the prognostic value of assessing *FOXK2* gene expression in HCC.^7^^,^^20^ Similarly, Lin et al demonstrated that FOXK2 served as an independent factor for overall survival, while FOXK2 silencing impaired cell growth and migration.^20^ The inhibition of migration in HCC cellular models was later confirmed by FOXK2 knockdown, which was associated with the modulation of epithelial–mesenchymal transition (EMT) markers, including E-cadherin and snail.^21^ Notably, high expression of FOXK2 has been associated with resistance to 5-fluorouracil *in vitro* and increased nucleotide synthetic gene expression in HCC patients.^7^ Finally, circular RNAs originating from the *FOXK2* gene have been shown to contribute to the Warburg effect and HCC progression *in vitro* and in animal xenografts.^22^ Altogether, these findings support the role of FOXK2 as an oncogene in HCC.

An oncogenic function has also been attributed to *FOXK2* in colorectal carcinoma (CRC). Consistent with our findings (AUC = 0.6734; *P* < 0.0001; Fig. 1B), The Cancer Genome Atlas (TCGA) and Oncomine datasets analyzed by other groups revealed that *FOXK2* gene expression was significantly higher in CRC than in the normal tissues.^23^, ^24^, ^25^ In particular, Du et al found that *FOXK2* was one of the most markedly increased FOX genes in CRC, with an up-regulated expression pattern in CRC and normal samples (adjacent non-tumorous and normal colorectal epithelial tissues).^23^ They also established an association of high FOXK2 protein levels with recurrence rates and shorter overall survival. Despite differences in patient cohorts and stratification, we found no statistical significance in addressing *FOXK2* expression for prognosis in our analyzed cohort of rectal adenocarcinoma patients (Fig. S2). When it comes to the molecular mechanisms involved, FOXK2 effects in CRC were associated with the EGFR/ERK,^23^ SOX9^24^ and Wnt/β-catenin signaling pathways,^25^ all of which are crucial for CRC development and/or progression. Consistently, Feng et al (2021) demonstrated that FOXK2 can bind to the vascular endothelial growth factor A (*VEGFA*) gene to promote angiogenesis and resistance to targeted therapy in thyroid cancer. This study found that *FOXK2* was up-regulated in the aggressive anaplastic thyroid carcinoma subtype, which was linked to tumor size.^26^ Although we did not observe any association between high FOXK2 transcript levels and overall survival in our cohort of patients (Fig. 2), a parallel study correlated the *FOXK2* gene and protein expression with clinical pathological parameters in papillary thyroid cancer, such as tumor size and Tumor Node Metastasis (TNM) stage.^27^ FOXK2 silencing impaired thyroid cancer cell proliferation *in vitro* through the increase in autophagic cell death, further providing mechanistic insights into the oncogenic functions of FOXK2. It is important to highlight that anaplastic and papillary thyroid carcinomas resemble distinct diseases with diverse clinical outcomes, which might reflect differences in *FOXK2* expression and prognostic impact.

Despite evidence in the literature on the oncogenic role and prognostic implications of *FOXK2* in HCC, CRC and thyroid carcinoma, *FOXK2* gene expression had no impact on overall survival in ESCA, cervical squamous cell carcinoma, ovarian cancer, pancreatic ductal, rectum and stomach adenocarcinomas; pheochromocytoma and paraganglioma, sarcoma, testicular germ cell tumor and thymoma to name a few (Fig. S2). This finding raises the possibility that assessing only the transcript expression of *FOXK2* might not be of clinical utility for most tumors. Corroborating this idea, the analysis of FOXK2 protein levels has been shown to provide clinical information in some tumors, such as breast cancer,^18^ gastric cancer^28^ and glioma.^29^ Interestingly, FOXK2 has been functionally validated as a tumor suppressor in all these tumor tissues, which might indicate that additional levels of gene regulation might contribute to delineating the biological functions of FOXK2. Of note, FOXK2 post-translational modifications have been increasingly reported as a mechanism of regulation of gene expression, including phosphorylation,^3^^,^^6^^,^^8^^,^^30^ SUMOylation,^31^ ubiquitination^32^ and acetylation.^11^ Therefore, it is likely that the mRNA levels might neither reflect the FOXK2 protein levels, *FOXK2* transcriptional activity nor biological function.

Although we found no clinical significance for assessing *FOXK2* transcript expression in most cancer tissues, this was not the case for ccRCC. We found reduced *FOXK2* gene expression in ccRCC tumor samples when compared to normal samples (*P* = 0.0076) (Fig. 1B). This finding corroborates a study that also found differential transcript expression of *FOXK2* in ccRCC and showed that *FOXK2* overexpression suppresses the epidermal growth factor receptor (EGFR) at both the protein and mRNA levels.^33^ Also, Kaplan–Meier curves showed that ccRCC patients with low *FOXK2* gene expression had significantly poorer disease-free survival (DFS) than patients with high *FOXK2* gene expression, which was revealed to be an independent prognostic factor, suggesting its role as a tumor suppressor in ccRCC.^33^ In contrast, our Kaplan–Meier curves showed that patients diagnosed with ccRCC with low *FOXK2* expression had better rates of overall survival than patients with high *FOXK2* gene expression (log-rank test *P* = 0.0021) (Fig. 2). Supporting our data, Jia et al^34^ have shown that low *FOXK2* expression was associated with longer overall survival and DFS in ccRCC patients, providing additional information as an independent prognostic factor in multivariate analysis. Similarly, Yang et al^35^ found that *FOXK2* was highly expressed in high-risk ccRCC patients. These differences in the role of *FOXK2* gene expression in ccRCC could be explained by the low number of samples analyzed in Zhang’s work,^33^ compared to cohorts extracted from large-scale datasets like TCGA^34^^,^^35^ and ICGC (International Cancer Genome Consortium).^35^

## FOXK2 gene methylation

**Figure 3:**
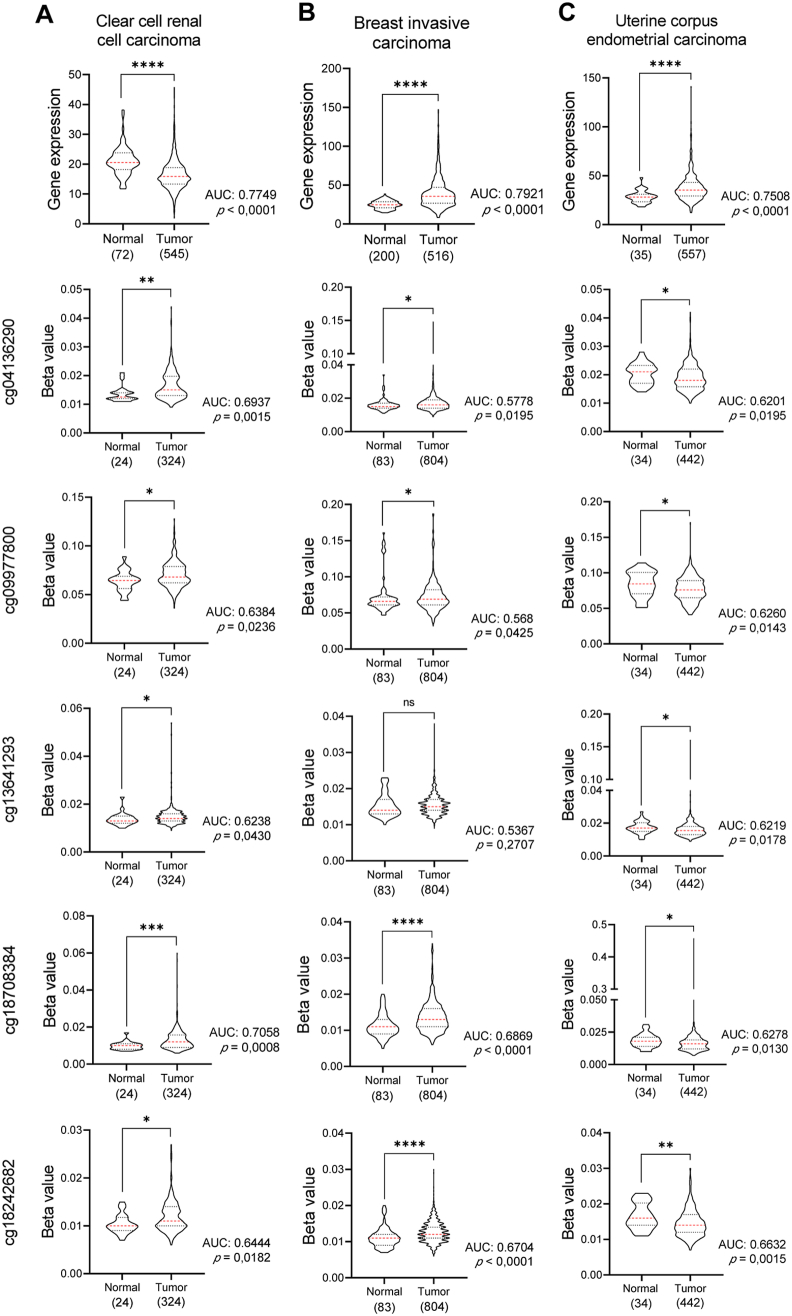
Gene expression and methylation status of *FOXK2* in different tumor types. *FOXK2* gene expression levels (first line graphs) were evaluated using RNA-seq data (TPM: Transcripts Per Kilobase Million) and *FOXK2* gene methylation status was assessed using the beta value of five probes that bind to the FOXK2 gene promoter (2–6 line graphs). Data were extracted from the OncoDB platform (Normal tissue samples: GTEx database; Tumor tissue samples: TCGA). The normal distribution of samples was evaluated via the D’Agostino & Pearson test and the cohorts were compared via the Mann–Whitney test. ns, non-significant; ∗*P* < 0.05; ∗∗*P* < 0.01; ∗∗∗*P* < 0.001; ∗∗∗∗*P* < 0.0001. For all analyses, the area under the curve value (AUC from the ROC curves) and the respective *P* values are highlighted in the graphs. Graphs were constructed with GraphPad Prism 8.0.1.

Considering that *FOXK2* transcript expression is variable across cancer types, we questioned whether methylation of the *FOXK2* gene promoter could play a role in regulating gene expression in different cancer tissues. For this purpose, we analysed five regions spanning the early region of the *FOXK2* promoter using the OncoDB interactive online cancer database available at www.oncodb.org.^36^ According to our analysis, the methylation status of the *FOXK2* promoter showed high variation across cancer tissues (Fig. 3 and Table 1). Samples from ccRCC patients are highly methylated in the *FOXK2* promoter compared to normal tissues. Accordingly, *FOXK2* expression levels are lower in tumors than in normal tissue samples (Fig. 3A). On the other hand, the *FOXK2* promoter is less methylated in UCEC than in normal tissues, while the *FOXK2* expression level is higher in these tumors than in normal samples (Fig. 3C). These data appear to reflect the classic repressive role of DNA promoter methylation in gene expression.^37^ However, BRCA (Fig. 3B) and LUSC (Fig. S3 and Table 1) samples presented higher *FOXK2* promoter methylation and higher *FOXK2* expression compared to normal tissue samples, suggesting that *FOXK2* promoter methylation could be associated with gene activation. For the other cancer types analysed in this review, two or more probes of *FOXK2* promoter methylation showed no significant difference between tumor and normal tissues, and little association was found between *FOXK2* promoter methylation and gene expression (Fig. S3 and Table 1).

**Table 1:** *In silico* comparative analyses of *FOXK2* promoter methylation in tumor *versus* normal tissues.

Tumor type (carcinoma)	Methylation status (OncoDB)					*FOXK2* gene expression		Prognosis
	cg04136290	cg09977800	cg13641293	cg18708384	cg18242682	OncoDB	TNM plotter	KM plotter
Bladder urothelial	*ns*	*ns*	*ns*	*ns*	–	+	+	*ns*
Clear cell renal cell	+	+	+	+	+	–	–	Favorable
Thyroid	*ns*	*ns*	–	–	–	+	+	*ns*
Uterine corpus endometrial	–	–	–	–	–	+	+	N/A
Breast invasive	+	+	*ns*	+	+	+	+	*ns*
Colon	+	+	*ns*	*ns*	*ns*	+	+	N/A
Esophageal	*ns*	*ns*	*ns*	*ns*	*ns*	+	+	*ns*
Head and neck squamous cell	*ns*	*ns*	+	+	+	+	+	*ns*
Kidney renal papillary cell	*ns*	*ns*	*ns*	*ns*	*ns*	+	+	*ns*
Hepatocellular	*ns*	*ns*	*ns*	*ns*	*ns*	+	+	Favorable
Lung adenocarcinoma	*ns*	*ns*	*ns*	*ns*	*ns*	+	+	*ns*
Lung squamous cell	+	+	+	*ns*	+	+	+	*ns*

The normal distribution of samples were evaluated by the D’Agostino & Pearson test. Data that follow a normal distribution were evaluated by Welch’s *t* test and data that do not follow a normal distribution were tested with Mann–Whitney test. Abbreviations: ns, not significant; probes, cg04136290, cg09977800, cg13641293, cg18708384, cg18242682; OncoDB, http://www.oncodb.org/; TNM plotter, https://tnmplot.com/analysis/; KM plotter, https://kmplot.com/analysis/. N/A: Not available. (+): High or (−): Low FOXK2 gene expression in tumor compared to normal tissues.

Consistent with that, increasing evidence that promoter hypermethylation may also be related to higher transcriptional activity in different contexts has been reported, including cancer.^37^^,^^38^ Although this phenomenon requires further investigation, some molecular mechanisms have been proposed, such as the recruitment of specific transcriptional activators and/or displacement of repressive transcription factors,^37^^,^^38^ interaction with distal regulatory elements and/or activity of alternative promoters.^37^ The *FOXA2* gene, another member of the FOX family, appears to be regulated in this manner during endoderm development,^37^^,^^38^ reinforcing the hypothesis that this may also be a regulatory mechanism of *FOXK2* gene expression. Although we highlight this hypothesis, we cannot exclude the possibility that other epigenetic mechanisms or post-transcriptional modifications also play an important role in regulating *FOXK2* gene expression. To date, studies have evaluated methylation at the *FOXK2* gene body,^39^, ^40^, ^41^ including the intronic regions.^42^ In a study involving morbidly obese patients, weight loss induced higher methylation and lower expression of *FOXK2*.^39^ This inverse relationship between methylation and expression has also been observed in T41A desmoid-type fibromatosis tumors.^40^ These data suggest that methylation in distinct regions of the *FOXK2* gene might play differential roles in repressing or activating gene expression. Therefore, the relationship between *FOXK2* promoter methylation and gene expression in different types of tumors should be further explored.

## FOXK2 gene alterations

We next explored the genetic mechanisms underlying *FOXK2* overexpression in cancer. For this purpose, we assessed *FOXK2* copy number and mutational status data in tumor samples from the TCGA repository through the Xena platform (https://xena.ucsc.edu/) and analyzed whether there was an association with *FOXK2* expression. For mutational status, patients were divided into wild-type and mutated groups (gene deletions and/or insertions and single nucleotide polymorphisms). For copy number variations, patients above the median were classified as high, while those below the median were classified as low. Notably, we observed low frequencies for *FOXK2* gene mutations, whether indels (gene deletions and/or insertions) or single nucleotide polymorphisms (SNPs) in cancer patients (Table 2, left panel). *FOXK2* gene mutations were found more frequently in bladder (2.2%) and colon (2.3%) cancer, while most tumors showed less than 1% mutation in the *FOXK2* promoter gene (Table 2, left panel). Except for HNSC, no significant correlation could be established between *FOXK2* expression levels and mutational status (Table 3, left panel). In contrast, *FOXK2* copy number variations have been shown more frequently, ranging from 28.9% gain of one or more alleles or segments in lung tumors to 1.5% in ccRCC patients (Table 2, right panel). When it comes to copy number loss, a 72.7% frequency was attributed to ccRCC, with only 0.2% attributed to thyroid cancer (Table 2, right panel). Interestingly, high levels of *FOXK2* were positively associated with high copy number alterations in all tumor types analyzed, except for THCA (Table 2; right panel). This suggests that *FOXK2* gene overexpression could be a result, at least in part, of *FOXK2* copy number variations. Although we pioneer evidence in cancer tissues, *FOXK2* copy number variations have been reported in human anorectal malformations^43^ and couples with recurrent spontaneous abortion,^44^ and further characterized in distinct pig breeds.^45^ Altogether, these findings indicate that chromosomal structural variations involving the *FOXK2* gene might be functionally significant and should be better explored in pathophysiology.

**Table 2:** Frequency of copy number variations and mutations in FOXK2 gene.

Carcinoma type	Mutation *status*					Copy Number (CN) variations^a^				
	*Wild type*	%	Mutated	%	Total	CN Gain	%	CN Loss	%	Total
Bladder	393	97.8	9	2.2	402	88	21.9	9	2.2	408
Breast	782	99.6	3	0.4	785	206	26.2	63	8.0	1080
Colon	259	97.7	6	2.3	265	90	20.0	55	12.2	451
Esophageal	182	99.5	1	0.5	183	41	22.3	28	15.2	184
Head and neck	489	99.2	4	0.8	493	79	15.1	56	10.7	522
Clear cell renal cell	361	99.7	1	0.3	362	1	1.5	48	72.7	66
Kidney papillary cell	276	98.9	3	1.1	279	36	6.8	22	4.2	528
Hepatocellular	348	99.4	2	0.6	350	89	24.1	5	1.4	370
Lung adenocarcinoma	491	98.8	6	1.2	497	149	28.9	16	3.1	516
Lung squamous cell	464	99.1	4	0.9	468	95	19.0	41	8.2	501
Prostate	487	99.8	1	0.2	488	9	1.8	5	1.0	492
Thyroid	485	99.6	2	0.4	487	22	4.4	1	0.2	499

a Copy number variation gain = gain of one or more alleles or segments; Copy number variation loss = loss of one or more alleles or segments.

**Table 3:** Association between FOXK2 gene expression and copy number variations or mutations in cancer patients.

Carcinoma tissue	*FOXK2* expression	Indel/SNP			Copy number		
		Mutated	*Wild type*		High	Low	
				*P* value			*P* value
Bladder urothelial				0.702			<0.0001
	High	4	200		153	50	
	Low	5	193		48	150	
Thyroid				0.156			7E-01
	High	2	241		121	122	
	Low	0	244		126	118	
Uterine corpus endometrial				0.986			<0.0001
	High	4	80		59	25	
	Low	4	79		25	58	
Clear cell renal cell				0.317			5E-03
	High	1	180		104	77	
	Low	0	181		77	104	
Breast invasive				0.553			<0.0001
	High	1	395		301	95	
	Low	2	387		92	297	
Colon adenocarcinoma				0.383			<0.0001
	High	2	133		97	38	
	Low	4	126		34	96	
Lung squamous cell				0.319			<0.0001
	High	3	232		184	51	
	Low	1	232		52	181	
Head and neck squamous cell				0.045			<0.0001
	High	4	243		180	67	
	Low	0	246		66	180	
Esophageal squamous cell				0.313			<0.0001
	High	1	90		63	28	
	Low	0	92		28	64	
Kidney renal papillary cell				0.076			<0.0001
	High	0	142		106	36	
	Low	3	134		34	103	
Hepatocellular				0.158			<0.0001
	High	0	174		128	46	
	Low	2	174		46	130	
Lung adenocarcinoma				0.634			<0.0001
	High	2	249		187	65	
	Low	3	242		63	182	
Prostate adenocarcinoma				0.313			<0.0001
	High	0	247		149	98	
	Low	1	240		95	146	

Indels refer to insertion and/or deletion of nucleotides into genomic DNA and include events less than 1 kb in length. SNP: single-nucleotide polymorphism.

## Concluding remarks and future perspectives

In this review, we systemically characterized *FOXK2* gene expression across a wide range of cancer types, with emphasis on how it can provide diagnostic and prognostic information of clinical utility. For some tumors, such as HCC, most studies point to a well-established oncogenic role for FOXK2,^7^^,^^20^, ^21^, ^22^ with high gene expression different from normal adjacent tissues and closely associated with poor prognosis.^7^^,^^20^^,^^22^ For others, additional layers of gene regulation seem to guide the biological roles of FOXK2 far beyond transcript expression, with post transcriptional and translational modifications emerging as crucial regulatory mechanisms to be considered. In breast cancer, FOXK2 protein overexpression inhibited cell growth^46^ and favored drug responses,^18^^,^^31^ with strong evidence indicating a tumor suppressive role in repressing carcinogenesis.^47^ FOXK2 protein dynamics contrast with high *FOXK2* mRNA levels and gene amplification in breast cancer, an issue worth exploring in future studies. Interestingly, *FOXK2* was validated as a direct target for microRNAs in other cancer tissues, such as HCC (miR 1271-5p and miR 122-5p),^20^^,^^48^ ESCA (miR-602)^49^ and non-small cell lung cancer (miR-1271),^50^ further suggesting that non-coding RNAs might modulate *FOXK2* expression and function. To add another level of complexity, the expression of alternative *FOXK2* splicing isoforms has not yet been analyzed and characterized in human tissues, which might be a confounding factor in addressing FOXK2 expression in cancer.

We herein also compiled work on the genetic alterations of the *FOXK2* gene, describing the frequencies of *FOXK2* genetic mutations and copy number variations in a wide range of tumor tissues. This is relevant considering that assessing the status of gene amplification and specific mutations of biomarkers has been increasingly established as useful diagnostic, predictive and prognostic information in cancer. Although a previous study has identified *FOXK2* mutations in cancer,^3^ it is likely that gene mutations might not be the major mechanism governing *FOXK2* gene expression. The same applies to promoter gene methylation, which we have shown to be poorly linked with *FOXK2* transcript levels. Nevertheless, the strong association between *FOXK2* copy number variations and *FOXK2* gene levels in most tumors represents a novel finding and highlights potentially relevant functional chromosomal abnormalities involving *FOXK2*. Of note, a partial tetrasomy 17q25.3 located within the *FOXK2* gene was reported for a child with malformation, severe development delay, and intellectual disability.^51^ Despite being a single report, we cannot exclude the possibility that disrupting the *FOXK2* gene at this breakpoint might have clinically important phenotypic consequences. Further validation in different and larger cohorts of cancer patients is needed to better establish the role of addressing *FOXK2* gene expression as a diagnostic, therapy prediction and prognostic factor in diseases, including cancer.

## CRediT authorship contribution statement

**Renata Ivo Vasconcelos:** Writing – review & editing, Writing – original draft, Investigation, Formal analysis, Data curation. **Luciana da Torre Carneiro:** Writing – review & editing, Writing – original draft, Investigation, Formal analysis, Data curation. **Raquel Ciuvalschi Maia:** Writing – review & editing. **Thaís Hancio:** Writing – review & editing, Methodology, Formal analysis, Data curation. **Gabriela Nestal de Moraes:** Writing – review & editing, Writing – original draft, Supervision, Resources, Project administration, Investigation, Funding acquisition, Formal analysis, Data curation, Conceptualization.

## Funding

Work conducted by G.N. was supported by grants from L’oreál-UNESCO-ABC For Women in Science, Carlos Chagas Filho Foundation for Research Support of the State of Rio de Janeiro (FAPERJ No. SEI-26003/004812/2021 and SEI-260003/001554/2022) and the National Council for Scientific and Technological Development (CNPq No. 307427/2022-6 and 408314/2021-4). R.I.V and L.T.C were supported by CNPq fellowships. R.C.M is supported by FAPERJ (No. 260003/007036/2022). Our laboratory was also supported by grants from the CNPq-INCT program (No. 408340/2024-0). The funding agencies have not been involved in the study design, data collection, analysis and interpretation or writing of the manuscript.

## Conflict of interests

The authors declare no competing financial interests.
