# Supplementary material for: FOXK2 gene expression in cancer: Potential regulatory mechanisms and clinical implications
Source: Genes Dis. 2025 Nov 24;13(4):101951. doi: 10.1016/j.gendis.2025.101951 (PMC13058959; doi:10.1016/j.gendis.2025.101951)
Supplement: Multimedia component 1 [file mmc1.pdf]

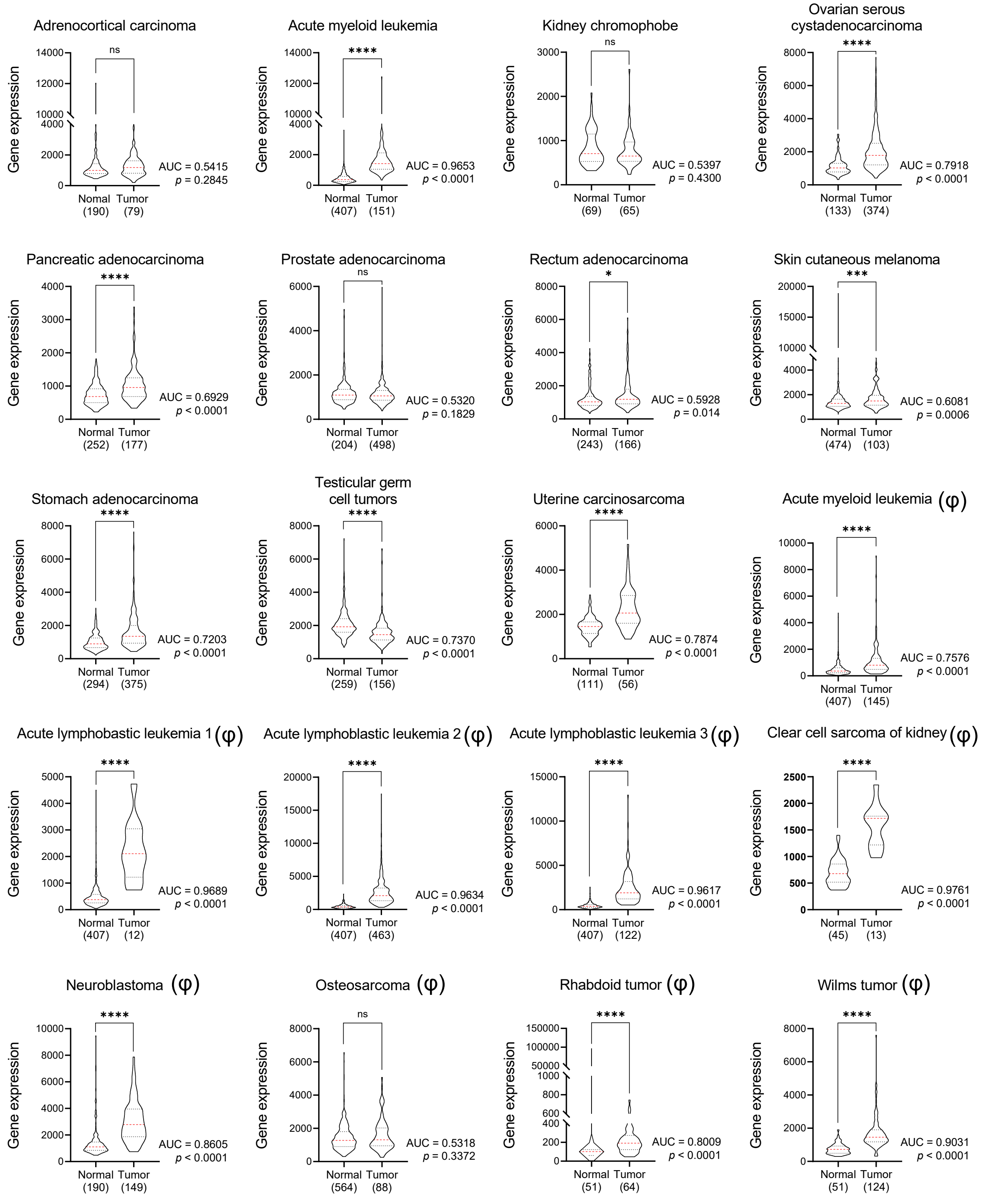

Supplementary Figure 1 - Expression of *FOXK2* gene in twenty different tumor types. Violin plots of *FOXK2* gene differentially expressed between normal and tumor tissue samples from 20 different tumor types. Tumor types marked with "φ" are from pediatric tumor datasets. The transcriptome profiling of the samples were processed by RNAseq technique. The normal distribution of the samples were evaluated by the D'Agostino & Pearson test and the cohorts were compared by Mann-Whitney test. Databases: TCGA (The Cancer Genome Atlas); GTEx (Genotype-Tissue Expression) and TARGET (Therapeutically Applicable Research to Generate Effective Treatments) for pediatric tumor tissues. ROC curves were analyzed and the area under curves value (AUC) and its respective *p* values are showed at the graphs. ns, not significant; **p*<0,05; ***p*<0,01; ***p<0,001; ****p<0,0001. Data were extracted from the TNMplot platform. Graphs were constructed with GraphPad Prism 8.0.1.

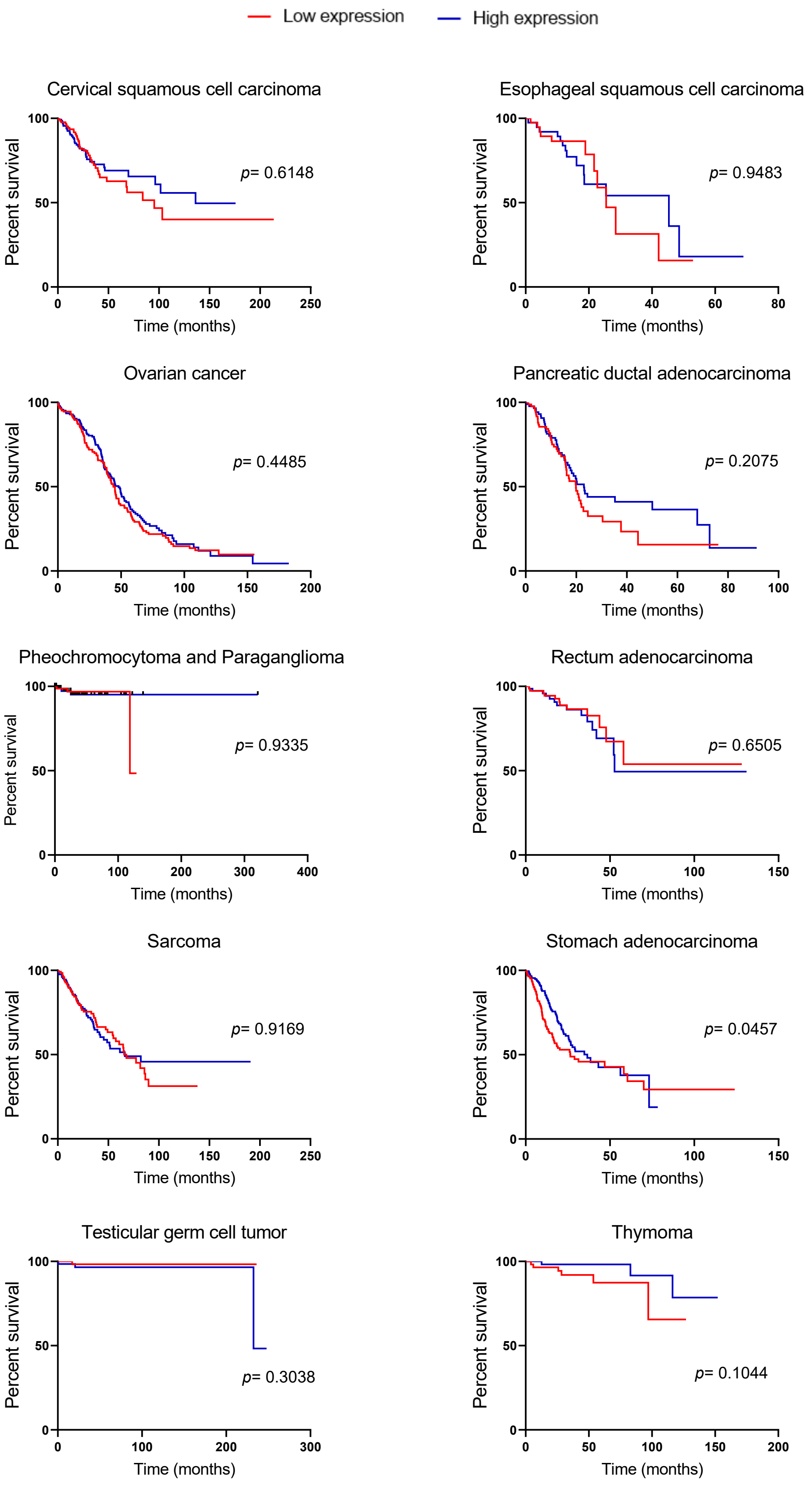

Supplementary Figure 2 - Impact of *FOXK2* gene expression on patient's overall survival in ten different tumor types. Correlation between Kaplan-Meier overall survival curves and low (red line) or high (blue line) *FOXK2* gene expression groups in different tumor types are plotted with its respectives log rank *p* values. *FOXK2* gene expression levels were evaluated using RNAseq data and the patients were splited by the median. Gene expression and overall survival informations are from GEO (Gene Expression Omnibus), EGA (European Genome-phenome Archive) and TCGA (The Cancer Genome Atlas) databases. The datasets were extracted from Kaplan-Meier plotter platform and the graphs and tests were made with GraphPad Prism 8.0.1.

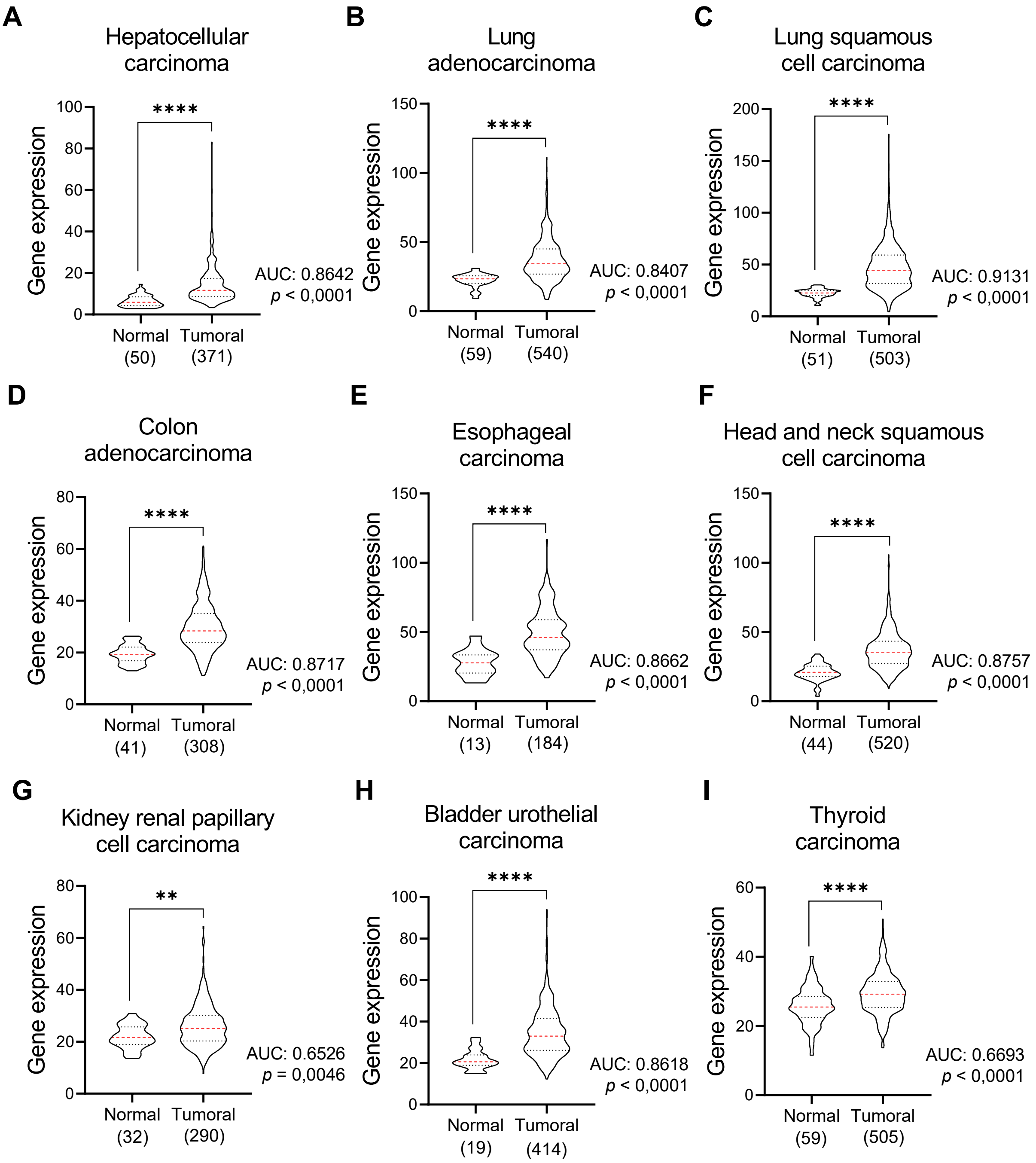

Supplementary Figure 3 – FOXK2 gene expression in different tumor types. *FOXK2* gene expression levels were evaluated using RNAseq data (TPM: Transcripts Per Kilobase Million) extracted from the OncoDB platform (Normal tissue samples: Genotype-Tissue Expression (GTEx) database; Tumor tissue samples: The Cancer Genome Atlas, TCGA). The normal distribution of samples were evaluated by the D'Agostino & Pearson test and the cohorts were compared by Mann-Whitney test. ***p*<0,01; *****p*<0,0001. For all analyses, ROC curves were made and the area under the curve value (AUC) and its respective *p* value are highlighted in the graphs. Graphs were constructed with GraphPad Prism 8.0.1.
